# Invasion-Associated Reorganization of Laminin 332 in Oral Squamous Cell Carcinomas: The Role of the Laminin γ2 Chain in Tumor Biology, Diagnosis, and Therapy

**DOI:** 10.3390/cancers14194903

**Published:** 2022-10-07

**Authors:** Alexander Berndt, Nikolaus Gaßler, Marcus Franz

**Affiliations:** 1 Section Pathology, Institute of Legal Medicine, University Hospital Jena, 07747 Jena, Germany; 2 Department of Internal Medicine I, University Hospital Jena, 07747 Jena, Germany

**Keywords:** laminin 332, gamma 2 chain, oral squamous cell carcinoma, invasion, extracellular matrix, fibronectin, tenascin-C

## Abstract

**Simple Summary:**

The destructive growth of carcinomas is associated with crossing the border between the epithelial and the connective tissue parts of an organ. One component of this borderline, the basement membrane, is the heterotrimeric laminin 332, which mediates the adhesion of basal epithelial cells. This protein, in particular its gamma 2 chain, is fundamentally reorganized during tumor cell invasion. Specific deposition patterns of laminin 332 are also present in oral squamous cell carcinomas and have been shown to be of high diagnostic and predictive value. Furthermore, laminin 332 restructuring is associated with important tumor biological processes, e.g., stromal activation, the development of a motile phenotype, and tumor spreading. In this review, current knowledge in the field is summarized and the recommendation to consider laminin 332 as a promising grading and monitoring parameter and as a potential therapeutic target is discussed.

**Abstract:**

Invasion of the connective tissue by carcinoma cells is accompanied by disintegration and reorganization of the hemidesmosomes, which connect the basement membrane to the basal epithelial cells. In terms of mediating the basement membrane, i.e., basal cell interactions, the heterotrimeric laminin 332 is the most important bridging molecule. Due to this distinct function, laminin 332, especially its gamma 2 chain, came into the focus of cancer research. Specific de novo synthesis and deposition patterns of laminin 332 are evident upon development and progression of oral squamous cell carcinomas (OSCCs). Loss from the basement membrane, cytoplasmic accumulation, and extracellular deposition are associated with crucial processes such as stromal activation and immune response, epithelial to mesenchymal transition, and tumor cell budding. In networks with components of the tumor microenvironment, altered expression of laminin 332 chains, proteolytic processing, and interaction with integrin receptors seem to promote cancer cell migration. Indeed, reorganization patterns are shown to have a high diagnostic and prognostic value. Here, we summarize the current knowledge on laminin 332 reorganization in OSCCs with special focus on its gamma 2 chain and provide, based on the current literature, evidence on its promising role as a grading and monitoring parameter and as a potential therapeutic target.

## 1. Introduction

Oral squamous cell carcinomas (OSCCs) represent the most abundantly occurring tumor type among head and neck carcinomas. As reported by the Global Cancer Observatory (GCO), there were 377,713 new cases and 177,757 deaths related to lip and oral cavity tumors worldwide in 2020 [1]. Although there are strong variations in the incidence among different regions and countries, oral cancer remains a big challenge for health care systems. Recently, great efforts have been made to further basic biological tumor research in order to understand the processes of invasion, progression, and metastasis formation, as well as to improve strategies for targeted and individualized therapies. Nevertheless, there have only been minimal changes in overall prognosis and survival rates, which have remained at appr. 40–50% [2].

The latter aspect is especially related to the lack of reliable markers for early diagnosis, prognosis, or therapy surveillance. In addition, there are no established molecular targets for individualized treatment approaches [2]. Therefore, there is an urgent need to establish such new diagnostic and prognostic biomarkers, as well as related molecular therapies.

The loss of the sessile epithelial phenotype and the gain of migratory capability is the first step in cancer cell invasion and a prerequisite for the formation of a metastatic behavior. This is necessarily associated with reorganization of the epithelial basement membrane to enable keratinocyte motility and phenotype transition. Loss of E-cadherin mediated cell–cell contacts and the disintegration of hemidesmosomes are known to play a pivotal role within this process. In hemidesmosomes of squamous epithelia, the integrity of the epidermal–dermal junction zone is mainly mediated by the interaction of laminin 332 (Ln332) and α6β4 integrin. Therefore, modulation of these hemidesmosomal components is strictly associated with squamous cell carcinoma development and progression. Furthermore, the subepithelial extracellular matrix has to be reorganized to allow cancer cell movement into the connective tissue [3,4].

With respect to the crucial role of the extracellular matrix (ECM) microenvironment for tumor cell behavior, understanding the ECM modulating processes at the invasive front may help to develop targeted pharmaceutical strategies to prevent cancer cells from spreading and encourage the improvement of therapeutic concepts for head and neck carcinomas [5,6,7]. Therefore, the aim of this review is to summarize current knowledge on the tumor biological importance of Ln332 and its interaction with fibronectin (Fn) and tenascin-C (Tn-C) as relevant components of the ECM in the development and progression of OSCCs.

## 2. ECM Reorganization in the OSCC Invasive Front

Invasion of carcinoma cells is a complex and multifactorial process including reorganization of cell–cell contacts, the acquirement of a mesenchymal-like phenotype, the degradation of the basement membrane, and a directed migration into the inherent surrounding tissue [8,9]. Interaction between the carcinoma cells and the components of the tumor microenvironment (TME), also known as tumor–stroma cross-talk, is essential for the initiation and maintenance of this process and includes the mutual activation of carcinoma cells themselves as well as stromal fibroblasts, endothelial cells, and inflammatory cells [10]. Additionally, in OSCCs, cytokine-mediated tumor–stroma cross-talk leads to the development of a desmoplastic stroma reaction dominated by activated fibroblasts which, among others, are responsible for the deposition of abundant amounts of collagens [11,12]. In that context, resident or attracted fibroblast precursor cells gain the alpha smooth muscle actin positive (aSMA^+^) myofibroblastic phenotype. In the last two decades, tumor myofibroblasts were frequently designated as cancer-associated fibroblasts (CAFs), which seems to be a simplification in the light of current knowledge [13,14,15]. Currently, it is widely accepted that CAFs are a phenotypically and functionally heterogeneous cell population depending on carcinoma type and exact localization [16,17]. Therefore, aSMA positive CAFs should better be designated as myCAFs to take their functionally important diversity into account [18,19].

Besides tumor–stroma cross-talk, the local reorganization of the ECM is another crucial step of OSCC invasion. The process of ECM reorganization includes proteolysis of the adjacent matrix structures, de novo synthesis of migration promoting matrix proteins, and a structural 3D organization of this novel quality ECM, which will be described in detail later [20,21]. As far as we know, both carcinoma cells and the cells of the TME contribute to this ECM reorganization within the basement membrane (BM) structures and the invasion front [22]. During the last three decades, evidence has been given that the reorganized ECM exhibits a lot of similarities to physiological and pathological tissue modulating processes, e.g., embryogenesis, wound healing, and fibrosis [23,24,25,26,27,28]. Therefore, this special ECM composition is also referred to as an “oncofetal” or “provisional” ECM (oncfECM or pECM) [29,30,31,32]. This ECM composition is characterized by the re-occurrence of certain molecular variants of extracellular adhesion proteins, which can be generated by alternative splicing of the pre-mRNA, alternative de novo glycosylation or chain assembly, and changes in the deposition pattern. These variants are expressed in early development but are virtually absent in healthy adult tissues [23]. With respect to cancer cell behavior in the invasion front, those extracellular adhesion proteins, in particular fibronectins, laminins, and tenascins, as well as their oncofetal variants seem to play a critical role within the modulation of cell–matrix interactions during the development of an invasive cell phenotype [33,34]. Re-expression of the oncofetal isoforms of these proteins modulates the ECM properties to enable and promote migration via new integrin-mediated cell–matrix contacts, new interactions with other ECM proteins, and the formation of guiding structures for cancer cell invasion [35].

In the 1990s, our group could already demonstrate a differential expression and reorganization of laminin isoforms and fibronectin variants in OSCCs associated with neoplastic transformation and invasion [24,36]. Since then, there has been growing evidence that reorganized oncofetal adhesion proteins may be of high prognostic and therapeutic importance in head and neck cancer. As discussed in detail later in this review, not only the occurrence of the newly formed matrix proteins themselves but also the molecular interaction and formation of multiprotein complexes, as visualized by colocalization studies [37], seem to influence the biological behavior of both oral cancer cells and oral CAFs. Furthermore, matrix reorganization modulates the inflammatory host response in HNSCC and therefore represents a putative target for immune therapy [38,39].

## 3. Laminin and Laminin Isoforms

Laminins (Lns) are constitutive components of the BM. The laminin (Ln) molecule is a heterotrimeric ECM adhesion protein, consisting of a large α chain and two smaller chains, β and γ. Up to now, eleven Ln chains encoded in the human genome have been described. Among them, there are five α chains (α1, α2, α3, α4, and α5), three β chains (β1, β2, and β3), and three γ chains (γ1, γ2, and γ3). Alternative splicing of the α3 chain leads to two transcript variants, the shorter α3A and the longer α3B chain. Because the α3A variant is primarily incorporated in the epithelial BM and the α3B chain seems to be preferentially expressed in the vascular BM of healthy tissue and has no association with cancer events [40], in the following we refer to α3Aβ3γ2 Ln as Ln332. 

Tissue and developmental specific gene expression, as well as molecular interactions, lead to intracellular heterotrimerization. While the α1 chain is expressed during epithelial morphogenesis and is only marginally detectable in few adult organs, the α2 chain is a component of the muscle basal lamina, the α3 chain is located in the BM of stratified epithelia, and the α4 chain is located in the vascular BM [41]. Until 2005, laminins were numbered in the order of their discovery [42]. The currently defined nomenclature of laminins is based on their chain composition [43]. The prototype of the Ln family proteins is Ln-111, composed of the α1, β1, and γ1 chain. It is the first described and, up to now, the best characterized Ln isoform [44].

Lns are organized in a cross-shaped manner. The C-terminus of the long alpha chain folds in five globular domains (L1–L5) responsible for the interaction of the molecule with the plasma membrane as a link between the BM and the intracellular filament system. Interaction is mediated by a high affinity to integrins, dystroglycans, and other proteins. The N-termini of the three Ln chains are organized separately. They include repetitive amino acid sequences, which are homologous to the epidermal growth factor (EGF) (also known as EGF-like repeats or LE motifs) with interspaced globular domains. The N-termini of most chains fold in a globular domain, the so-called Ln N-terminal domain LN. The N-terminus is involved in molecular interaction with the collagen IV network in the BM. Furthermore, Lns with three full-length short arms are capable of self-polymerization. These polymers are connected, via perlecan and nidogen (entactin), to collagen IV polymers forming large networks crucial for BM formation. For detailed information concerning the molecular structure and general characteristics of laminins, see the excellent reviews by M. Aumailley and H. Colognato [41,45,46].

## 4. Ln332 and Hemidesmosomes

**Figure 1:**
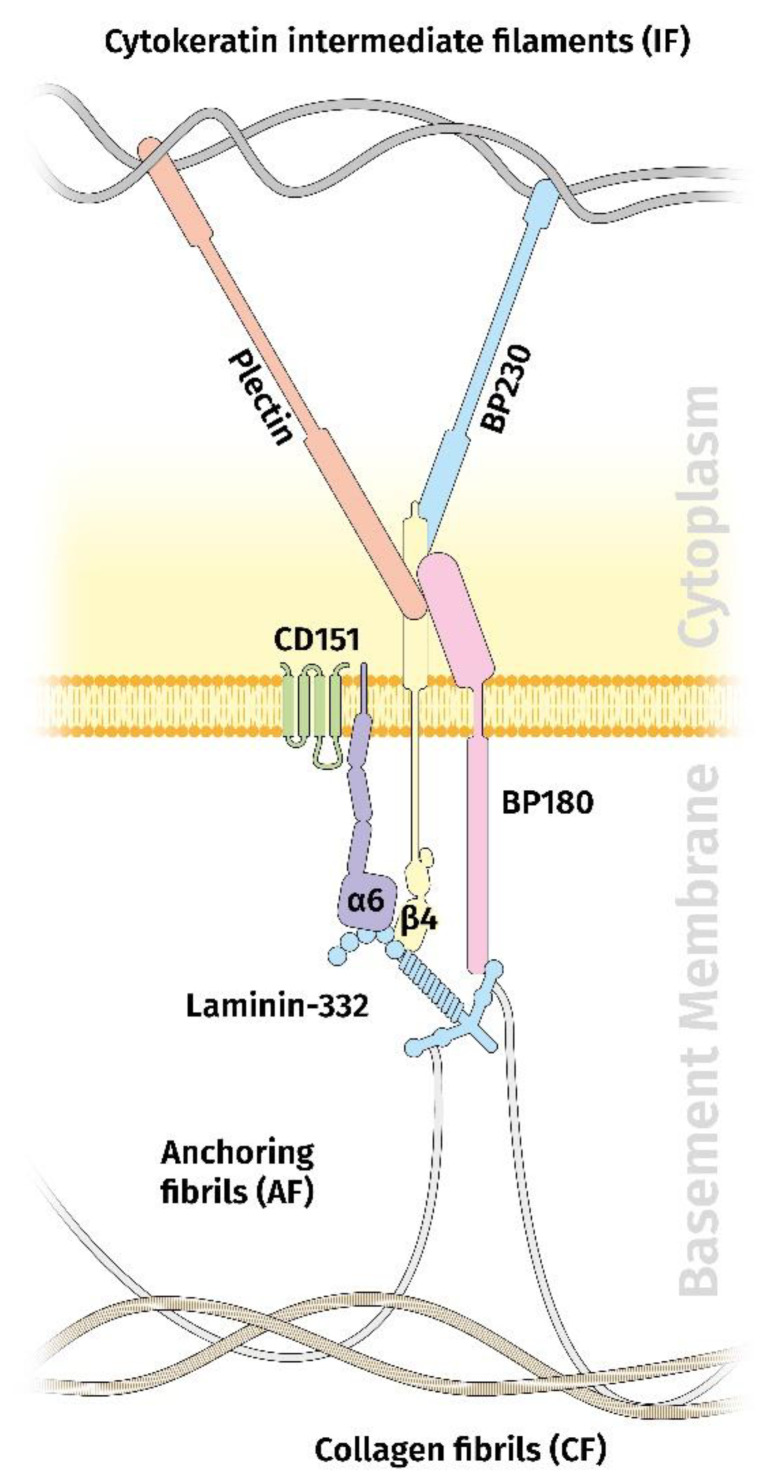
Schematic diagram showing the organization of the hemidesmosme in the dermal–epidermal junction. Besides laminin 332, the hemidesmosome contains the integrin α6β4 receptor, BP180, CD151, BP230, and plectin.

The functionally most important laminin within the dermal–epidermal junction is the laminin variant 332 (Ln332), formerly called laminin-5 [42,43]. Along with its molecular interaction partners and the integrin receptor α6β4, it represents the main component of the extracellular part of the hemidesmosome (HD). HDs are crucial for the maintenance of tissue integrity at the mesenchymal–epidermal contact zone. There are two types of HDs, type I (HD I) and type II (HD II), with HD I as the most common one in stratified and pseudostratified epithelia, including the epidermis and the airway epithelium. Besides Ln332, HD I is composed of α6β4 integrin, the bullous pemphigoid antigen (BP) 180 (also known as collagen type XVII), the tetraspanin CD151, and, inside the cell, BP230 and plectin [47] (Figure 1).

HD dynamics are crucial for tissue modulating processes in the dermal–epidermal junction, as seen in embryogenesis, organogenesis, wound healing, and, finally, in carcinoma cell invasion and migration. HD dynamics and related cell signaling is mainly regulated by α6β4 [48]. Interestingly, Ln332 plays a critical role in the formation and turnover of HDs. It is synthesized by keratinocytes and secreted in its unprocessed form. Extracellular proteolytic processing of its α3 chain occurs and enables the interaction with α6β4 integrin in stable HDs of quiescent epidermis. Additional processing of the γ2 chain is known to generate a 105 kDa product. When keratinocytes or carcinoma cells become motile (e.g., skin wounding or invasion), unprocessed Ln332 (α3 chain) seems to be secreted at the leading edge, enabling cell movement through the α3β1 integrin [49]. In this case, further processing of the γ2 chain is also known and will be described later.

With respect to its function as a binding partner to α6β4 or α3β1 integrins and the extracellular matrix network, Ln332 is involved, first, in HD formation and, second, in both inside-out and outside-in signaling. On the other hand, HD remodeling must be also reflected by altered Ln332 synthesis, secretion, and deposition pattern [49]. Therefore, Ln332 came into the focus of cancer invasion and metastasis research once the molecule was described and characterized. Indeed, BM modulation, as detected mainly by Ln332 immunohistochemistry, was reported for many carcinoma types and was characterized by loss of Ln332 from the BM zone accompanied by a cytoplasmic accumulation, as well as by extracellular deposition outside the BM region [50,51]. The tumor-promoting role of Ln332 was also described for different squamous cell carcinomas, and the molecular background behind this was recently summarized by P. Marinkovich [52]. Such changes were also detected in HNSCC, especially in oral squamous cell carcinomas, both in vitro and in vivo, which will be now described in more detail.

## 5. Laminin Reorganization in OSCCs

In the 1980s, Ln expression was already being investigated in OSCCs in relation to BM structure, showing intact BM staining in normal and hyperplastic epithelium but a loss of laminin in dysplastic oral mucosa and, especially, in invasive tumor regions [53]. Attenuation or loss of Ln staining in the BM zone was shown to be associated with a higher grade of malignancy [54] and, later, it became evident that loss of BM material (as shown by Ln immunohistochemistry) was also related to the neoplastic transformation process [55] and to an enhanced risk of regional lymph node metastasis [56]. Furthermore, immunohistochemical assessment of ECM molecule expression including Ln was postulated to be helpful in improving the grading of OSCCs [57,58]. Also at this time, several studies demonstrated that Ln BM reorganization was accompanied by a restructuring of the ECM proteins collagen type IV, fibronectin, and tenascin in the invasion front [57,59]. Additionally, during the late 1980s and the 1990s, several members of the Ln family were discovered and specific antibodies became available, such that investigations concerning Ln in the BM of oral mucosae were more and more focused on different Ln isoforms. In 1992, Epiligrin, currently known as Ln332, was described as the major keratinocyte integrin ligand involved in bullous skin disease as a target for autoantibodies and it became evident that this Ln isoform was the major BM Ln in squamous epithelia [60].

Years before, homologue proteins were described by several groups and named as BM600/nicein, GB3 antigen, kalinin, and ladsin (for review see [50]), which, in the end, mean the same molecule, which is Ln5 according to the unified nomenclature as suggested by Burgeson and coworkers [42]. According to the fact that ECM proteins have a crucial impact on cellular differentiation, proliferation, and migration [23], and that Ln5 was the major laminin of the squamous epithelial BM, research became focused on elucidating differential expression and changes in spatial distribution during neoplastic transformation, invasion, and tumor progression in OSCCs. Altered synthesis and deposition of Ln5 was already described in 1997 by Kainulainen and coworkers, demonstrating increased cytoplasmic immunostaining along with Ln γ2 chain (Lng2) mRNA detection in carcinoma cells at the invasion border [61]. In 1999, our group published, for the first time, a comprehensive analysis of the immunohistochemical expression of different Ln chains in oral mucosae and OSCCs [24]. Staining was performed using chain-specific antibodies on shock-frozen tumor samples. According to the results, the BM of normal adult oral epithelia comprises the α3, α5, β1, β3, γ1, and γ2 chain. In hyperproliferative conditions, and during neoplastic transformation, the α2 and the β2 chain were re-expressed. An increased expression of the β3 and γ2 chain at the invasive front was associated with cytoplasmic accumulation in budding carcinoma cells, as well as deposits outside the basement membrane region. Based on these findings, we suggested that Ln5 is an immunohistochemical marker for invasion and OSCC cell invasion, which is guided by the reorganized Ln5 matrix. Furthermore, α2 and β2 chain re-expression hints to a more embryonal state of the BM zone during the epithelial reorganization process. In line with increased cytoplasmic accumulation in tumor cells and extracellular depositions, there was a total loss of Ln5 from the tumorous BM. Quantification of Ln5 loss by confocal laser scanning microscopy correlated with the malignancy grade [62].

A BM-independent reorganization of Ln5 could also be proved in 3D cell culture models. In 1989, Matsumoto and coworkers presented, for the first time, a collagen gel-based in vitro model for OSCCs, demonstrating that gel-incorporated fibroblasts are necessary for the invasive behavior of tumor cells [63]. Accordingly, our group and others could immunohistochemically demonstrate a deposition of at least the Ln α3 and γ2 chains in the tumor–gel interface without the formation of a structural BM or hemidesmosomes [36,64].

OSCC invasion and progression is not only accompanied by a modulation of Ln chain expression in the tumor BM, but also in the tumor microenvironment. myCAF occurrence and endothelial activation seem to be associated with a decrease in α2 and an increase in α3, α4, α5, and γ2 chain expression in the stromal compartment, as well as modulation of α3 chain expression in tumor vessels. Interestingly, γ2 chain positivity was also seen in some vascular structures in well-differentiated OSCCs [65].

## 6. Laminin γ2 Chain Reorganization in the OSCC Invasive Front: Tumor Biological Implications

As stated before, the γ2 chain is unique for Ln5/Ln332. Therefore, most investigations concerning the Ln332 reorganization in OSCCs that have been performed considered this chain as an immunohistochemical surrogate for Ln332, using different antibodies specific for the molecule. Indeed, there are several reports on the diagnostic and predictive value of Lng2 immunohistochemistry in OSCCs. Among others, a correlation has been shown between a diffuse expression of Lng2 in disseminating and infiltrating tumor cells, with higher grades of malignancy, poor prognosis, and a higher risk for nodal metastasis observed in patients with tongue cancer [66,67,68,69]. Furthermore, shorter life expectancy in OSCCs with an increased number of Lng2-positive tumor cells [70], an elevated risk for transformation in Lng2-positive pre-neoplastic mucosal lesions [71,72], as well as a higher risk for nodal metastasis [20,73,74] could be evidenced. Interestingly, expression of Lng2 in invading human carcinoma cells was already described as a common phenomenon in 1994 by C. Pyke and colleagues [75,76] and has been repeatedly demonstrated for several other epithelial tumor entities. Table 1 provides an overview of the relevant literature describing the impact of Lng2 assessment on OSCC diagnosis and prognosis. 

Against the background of these clinico-pathological findings, a link between altered Lng2 synthesis/deposition and the tumor biological behavior of neoplastic oral keratinocytes must be assumed. Although it cannot be completely excluded that the tumor expression pattern of Lng2 is merely a secondary phenomenon following dysregulation of laminin turnover during neoplastic transformation, disturbed BM formation, and/or phenotype transition, there are credible arguments for a direct protumorigenic influence of this laminin chain on cellular activity, which now will be discussed.

### 6.1. Laminin γ2 Chain Expression Is Related to the Migration of Normal and Neoplastic Keratinocytes

Lng2, probably in its monomeric form, seems to play an important role in guiding keratinocytes during wound re-epithelialization [81,82]. For a review of the contributions of an ECM to skin wound healing, see the excellent reviews of P. Rousselle [83,84]. This process was also proven to be crucial in an in vitro wound healing assay using rat oral epithelial cells. Here, accumulation of Lng2 was detected in the peripheral cytoplasm of cells at the wound edge, with migration mediated via the integrin α3 chain [85]. Ln332–α3β1 integrin interaction has been proven to direct the stabilization of polarized lamellipodia in epithelial cells via activation of Rac1 [86,87]. Alternatively, Decline and Rousselle described an α2β1-dependent mechanism of keratinocyte migration in human foreskin keratinocytes based on interaction with the short arm of Lng2 in unprocessed Ln332 [88]. The putative role of Lng2 in guiding keratinocyte migration during physiologic morphogenesis is also supported by the fact that the molecule shows a differential expression pattern and developmental change in early human embryonic and fetal tissue [89].

Although there are some differences in the deposition and proteolytic processing of Lng2 between normal and neoplastic tissue, squamous carcinoma cell migration seems to mirror keratinocyte movement during wound healing and vice versa. Not surprisingly, there are also several reports on the migration promoting activity of Ln332 or Lng2 for carcinoma cells in vitro and in vivo. This seems to be a common phenomenon that is detectable in cell lines of different carcinoma entities and is communicated as being associated with epithelial to mesenchymal transition (EMT) and the EGFR pathway [90,91,92]. Additionally, for OSCCs, a relation between Lng2 expression and cancer cell motility, along with a regulatory effect of different miRNAs or long non-coding RNAs, has been assessed [93,94]. Additionally, there seems to be a relation between Lng2 expression in carcinoma cells, EGFR signaling, and EMT. Although we were not able to immunohistochemically evidence a correlation between activation of EGFR downstream signaling (detected by using phosphor-specific antibodies against p44/42MAP kinase, p38 MAP kinase, and Akt) and invasion-associated accumulation of Lng2, a relation to EGFR expression could be proven by others for esophageal SCC in situ and in vitro [95,96]. Accordingly, we were able to show that stimulation of OSCC cells in vitro with EGF and TGFβ1 causes mesenchymal trans-differentiation accompanied by increased synthesis and deposition of Lng2, as well as raised invasive capability [97]. This goes in line with the findings of Ono and colleagues which showed a correlation between EGFR amplification and Lng2 protein expression in OSCC cell lines [98]. Additionally, Lng2 protein expression seems to be associated with a migratory EMT phenotype of carcinoma cells in situ [99]. Interestingly, the full EMT phenotype, as induced by SNAIL transfection of OSCC cells, is associated with a lack of Ln332 expression, probably caused by an inhibition of α3 chain synthesis but unchanged γ2 production. Therefore, accumulation of Lng2 seems to be a marker for the ongoing mesenchymal transition of OSCC cells [100].

### 6.2. Proteolytic Processing of Laminin γ2 Generates Migration Promoting Matrikines

**Figure 2:**
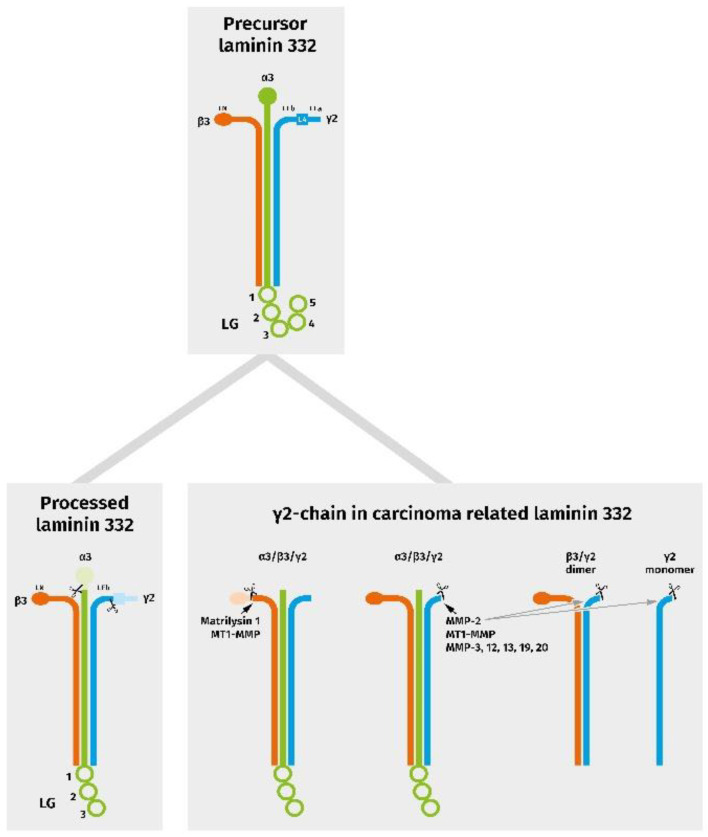
Schematic diagram representing chain assembly and proteolytic events in physiologically processed, as well as in carcinoma-related, laminin 332 variants. In tumors, the γ2 chain can be present in association with the α3 chain and a truncated β3 chain, as well as with a further specific cleavage in a heterotrimeric, dimeric, and/or monomeric form.

As stated above, the biological activity of Ln332 and its single chains is strictly associated with and determined by proteolytic processing. Proteolytic degradation of ECM structures in the invasive front is a precondition for carcinoma cell invasion into preexisting healthy subepithelial tissue; however, it is more than a pure destruction [22,101]. Thus, ECM degradation prepares the grounds for an embryonal extracellular reorganization and re-expression of the oncofetal variants of different matrix proteins, providing the necessary microenvironmental flexibility for tissue remodeling. Furthermore, proteolytic processing leads to newly structured 3D protein networks with altered binding characteristics, the solubilization of growth factors, and the liberation of bioactive fragments. With respect to Ln332, matrix metalloproteinases (MMP) play a pivotal role [51]. Lng2 processing during tumor progression and the induction of migration seems to be mostly associated with MMP2 and MT1-MMP [102,103], but MMP-3, -12, -13, -19, and -20 may also be of importance [51]. In humans, MT1-MMP cleavage leads to the generation of a 100 kDa fragment, γ2′, and a 85 kDa fragment, γ2x, as well as two smaller DIII fragments [104]. Comparable cleavage patterns have also been observed in mice [105]. The generation of further, functionally relevant Lng2-derived fragments during keratinocyte or carcinoma cell migration cannot be excluded. In addition to the Lng2 chain, proteolytical processing of the β3 chain by MT1-MMP and/or Matrilysin 1 also seems to be associated with increased cell motility [106,107] (Figure 2).

Additionally, for OSCCs, an association between Lng2 cleavage and the presence of MMP-2 and MT1-MMP, and their regulation via extracellular calcium, could be shown in vitro [108,109]. This is in line with reports on a correlation between immunohistochemical MMP2 and/or MT1-MMP expression and OSCC progression and metastasis [110,111] (for review see [112]). Furthermore, combined immunohistochemical evaluation of Lng2 and MT1-MMP has a predictive value for cervical lymph node metastasis in squamous cell carcinomas of the tongue and floor of the mouth [74]. Interestingly, although it was originally thought that MMPs were synthesized by the tumor cells, it becomes evident that these enzymes are also abundantly expressed by surrounding CAFs. In line with this, we were able to demonstrate coordinated immunohistochemical and mRNA expression of Lng2, as well as a spatial association with TGFβ1 and MT1-MMP/BMP1 in the stroma of the OSCC invasive front. Here, the stromal Lng2-positive cells displayed a mesenchymal phenotype, as shown by vimentin positivity, suggesting that either activated CAF or EMT-like OSCC cells contribute to the deposition of processed promigratory Lng2 [99]. However, it should be mentioned that plasmin may also have an impact on degradation and impair BM assembly of Ln332 [113].

Recently, our understanding of ECM–cell interaction has changed from a passive role towards a more dynamic mutual interaction, whereby ECM components exert signals strongly influencing cell differentiation, proliferation, and tumor biological behavior [114,115]. Besides the common haptokine signaling via integrin/adhesion receptors, ECM proteins can also harbor “cryptic” ligands that are exposed or released during conformational changes after proteolytic processing (also known as matrikines or matricryptins) [116]. Prototypical matrikines involved in epithelial/cutaneous processes such as wound healing or tumor cell invasion include the epidermal growth factor-like repeats (EGF-L) of tenascin-C and laminin/Lng2. In contrast to normal growth factors, EGF-L binds to the EGFR with a low affinity/high dissociation constant [117]. It is suggested that these matrikines signal to various cells in a distinctly promigratory way without inducing significant proliferation [118]. Although this hypothesis is proven for several epithelial tumors in vitro, there are nearly no reports for oral cancer. However, supporting this hypothesis, Fullar and colleagues showed that carcinoma cell–CAF interaction regulates MMP and tissue inhibitor of metalloproteinase (TIMP) synthesis in OSCCs [119]. We recently demonstrated that the secretome of activated fibroblasts is able to induce MMP activity in the membrane of OSCC cells, thereby releasing ligands (so far not defined in detail) that activate EGFR and subsequently induce EGFR upregulation. Furthermore, there is a stimulation of the highly oncogenic hetero-dimerization between HER3 and p95HER2 [120]. These results give further strong evidence that the TME influences the regulation of Ln332 reorganization and modulation of its tumor biological activity.

Besides this more local paracrine regulation of growth factor receptor activity, another route of Lng2 action has to be taken into account here. Thus, it could be shown that Lng2 can also be delivered via OSCC cell-derived extracellular vesicles inducing in vitro lymphangiogenesis by integrin-dependent uptake in lympho-endothelial cells [121], supporting a potential mechanism of mediating nodular metastasis.

### 6.3. Modulation of Laminin γ2 Expression Pattern Is a Result of Tumor–Stroma Cross-Talk and EMT

**Figure 3:**
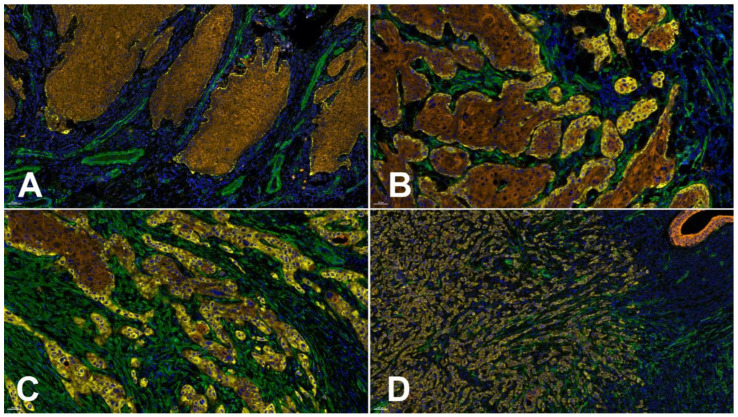
Different laminin gamma 2 chain (Lng2) deposition patterns in relation to the mode of invasion and the presence of myCAFs demonstrated by 4plex immunofluorescence staining using the Opal Multiplex Detection System from AKOYA Biosciences (full protocol available on request). The antibodies used were: clone B2 against Lng2 (Sc-25341, Santa Cruz Biotechnology, Inc., Heidelberg, Germany, yellow), clone 1A4 against alpha smooth muscle actin (aSMA) (IR611, Agilent Technologies Germany GmbH & Co. KG/DAKO, Hamburg, Germany, green), and clone AE1/AE3 against pan Cytokeratin (IR053, Agilent Technologies Germany GmbH & Co. KG/DAKO, Hamburg, Germany, brown). DAPI was used for nuclei counterstaining (blue). (**A**) OSCC with pushing well delineated infiltrating borders,, no aSMA-positive stromal fibroblasts (myCAFs), histopathological grade G2, a budding score of 1 according to the International Tumor Budding Consensus Conference (ITBCC 2021 [129]), and minimal basement membrane (BM) decoration for Lng2. (**B**) An OSCC showing infiltrating solid cords, clusters of myCAFs, histopathological grade G2, a budding score of 3 according to the ITBCC, and cytoplasmic accumulation of Lng2 in a few invasive disseminated OSCC cells in addition to positive marginal cells in more central parts of the tumor. (**C**) An OSCC with small groups or cords of infiltrating cells, myCAFs present in the whole invasive front, histopathological grade G3, a budding score of 3 according to the ITBCC 2021, and up to 50% Lng2-positive OSCC cells. (**D**) An OSCC showing a widespread dissemination of single tumor cells or small groups, an abundant presence of myCAFs, histopathological grade G3, a budding score of 3 according to the ITBCC 2021, and predominant Lng2-positive OSCC cells. Bar = 50 µm.

It is well known that the interaction of carcinoma cells with TME cells is a prerequisite of cancer cell invasion and tumor progression [122]. Using in vitro 3D invasion models, it could be impressively shown that the presence of fibroblasts is mandatory for the development of an invasive OSCC cell phenotype, and that this is also accompanied by Lng2 synthesis and deposition [36]. This is in line with several reports postulating the presence of myCAF in OSCC tissue as a marker for more aggressive tumor behavior and thereby as a predictor of cancer progression and prognosis [123,124,125]. The tumor-promoting activity of myCAFs seems to be associated with the induction of EMT. This could be shown in vitro for OSCCs, as well as tongue SCCs [126,127], and was evidenced by comparative immunohistochemical analyses of the expression of aSMA and EMT markers in situ in tongue SCCs [128]. Furthermore, EMT seems to also be related to the phenomenon of tumor budding. Tumor budding is defined as the presence of single cancer cells or clusters up to <5 cells in the invasive front. Budding can be scored according to the guidelines of the International Tumor Budding Consensus Conference [129], and is also reported as a prognostic indicator for OSCC [130,131,132]. The quantification of Lng2-positive buds was considered as a prognostic and predictive marker in different carcinoma entities, including HNSCC [133]. With respect to these findings, the direct influence of myofibroblastic stroma on Lng2 reorganization and associated EMT must be assumed. As far as we know, the molecular basis of this interrelationship is currently not investigated and understood in detail. The supply of enzymes for proteolytic Ln332 processing, the delivery of growth factors influencing tumor cell phenotype and metabolism, and active ECM organization seem to play a central role. Supporting this hypothesis, it was recently be shown that there is indeed a relation between the extent of budding, the presence of myCAFs, and Lng2 expression in OSCCs [79]. Examples of different immunohistochemical Lng2 expression patterns in relation to the presence of myCAFs are given in Figure 3.

### 6.4. Laminin γ2 Interacts with Oncofetal Fibronectin and Tenascin-C

As we know from re-epithelialization during wound healing, migration of keratinocytes is also regulated by an orchestrated remodulation of the ECM composition within the wound bed in space and time [84]. This includes not only a reorganization of laminin itself, but also the reoccurrence of special variants of cellular fibronectin (Fn), tenascin-C (Tn-C), and others. Isoforms of cellular Fn and Tn-C generated by alternative splicing of so-called extra domains, or by de novo glycosylation, are re-expressed during tissue remodeling processes and are only rarely detectable in healthy adult tissues. Cellular Fn variants containing the extra domains (ED) A and B (EDA-Fn, EDB-Fn), as well as domains in the IIICS region, are known to be associated with embryogenesis, wound healing, angioneogenesis, and carcinoma invasion [134,135]. Therefore, they are also known as oncofetal Fn variants (oncfFn). It has been shown that stromal fibroblasts and endothelial cells are mainly responsible for the synthesis of this molecular variant in OSCCs [24,136]. Interestingly, cellular Fn is able to modulate migration modalities (collective versus single cell) of OSSC cells in vitro in a differentiation-dependent manner [137]. Additionally, the hexameric glycoprotein Tn-C, a member of a protein family comprising at least four different molecules in humans, is a multidomain protein with up to nine fibronectin type III-like repeats (FNIII), which can be omitted or included by alternative splicing of pre-mRNA. As with Fn, large Tn-C isoforms are also known to be re-expressed during tissue modulating processes such as wound healing, inflammation and fibrosis, angioneogenesis, and in the carcinoma invasive front (oncfTn-C) [138].

Dependent on the individual splicing pattern, oncfTn-C is able to develop various effects in relation to tumor development and progression [139]. It is well known that oncfTn-C is able to modulate tumor cell behavior and inflammatory reaction in a tumor-type-dependent manner [140]. There are also several reports on the tumor biological, diagnostic, and prognostic relevance of oncfTn-C in OSCCs [68,141,142,143]. Hindermann and coworkers were able to show that the main sources of large unspliced Tn-C variants in OSCCs are invasive cancer cells. Stromal fibroblasts also contribute to its mRNA synthesis [144]. It has been shown that the protein is deposited in the stroma in close proximity to the invasive front, and that the extension of mRNA synthesis correlates to the grade of malignancy. With respect to the overlapping expression and deposition patterns of Lng2, oncfFn, and oncfTn-C, a structural interaction of these molecules may be assumed. In 1998, Ramos and coworkers had already shown in vitro that extracellular fibrillary organization of TnC depends on the presence of fibroblasts and fibrillary Fn, indicating a direct interaction between these matrix proteins [145]. Later, our group were able to prove that OSCC cell-produced Lng2 is also fibrillary organized in vitro in the presence of fibroblasts and is colocalized with fibrillary Fn and Tn-C [37]. This colocalization pattern could also be confirmed in situ in the invasive tumor front [146]. Although the functional relevance of the structural 3D organization of these matrix multiprotein complexes is not yet fully understood, it seems to play a crucial role in tumor biology. It may be assumed that the oncofetal restructuring of extracellular multiprotein complexes at least modulates physical properties for tissue modulation and morphogenesis. This hypothesis is supported by results from laser scanning microscope studies on OSCCs, demonstrating increased oncfTn-C incorporation and colocalization with Lng2 in the invasive front of the BM region with rising malignancy grade [147]. This may represent a step toward the higher flexibility of the BM at the tumor–stroma interface and a prerequisite for BM disintegration during OSCC progress. Furthermore, it was suggested that fibrillary multiprotein complexes containing oncfTnC, oncfFn, and Lng2 form tracks guiding cancer cell invasion and the movement of endothelial and inflammatory cells, as well as fibroblasts [148]. In line with these hypotheses, it could be shown that stromal co-expression of Fn and TnC is a strong prognostic marker in tongue carcinomas [149]. The biological importance of oncofetal extracellular multiprotein complexes for tissue remodeling and morphogenesis is additionally supported by the fact that, during tumor angioneogenesis, sprouting new vessels are also surrounded by a stratified oncfFn and onfTnC matrix [150].

## 7. Conclusions

In this review, we have summarized the current knowledge on the remodeling of Ln332 during OSCC development, invasion, and progression. Reorganization of hemidesmosomal contacts in the invasive front of OSCCs leads to aberrant synthesis, processing, and deposition of Ln332 and its chains, mainly caused by tumor-specific proteolytic patterns and tumor–stroma cross-talk.

Remodeling in the invasive front shows a variety of similarities to re-epithelialization during wound healing. Ln332 reorganization is critically modulated by OSCC–myCAF interaction, whereas cancer fibroblasts are responsible for the proteolytic landscape, synthesis, and deposition of further oncofetal matrix proteins, as well as 3D organization of the invasive front of an ECM.

The most remarkable cancer-associated deposition pattern is the abundant accumulation of Lng2 in the cytoplasm of invading tumor cells in the invasive front. This pattern is associated with an EMT-like phenomenon and is of relevant diagnostic and prognostic value. Cytoplasmic Lng2 accumulation is furthermore associated with tumor cell budding, which is a potential additional grading parameter. Up to now, invasive front grading has not yet been introduced in the WHO grading system for OSCCs; however, there is increasing evidence that the distribution pattern of Lng2-positive OSCC cells predicts patient outcome and may play a key role in optimizing therapy and the development of novel therapeutic strategies. In line with this, Lng2 immunohistochemistry has been reported to improve diagnosis in tissue sections, as well as the value of oral brush cytology [77,78]. Furthermore, serum concentrations of Lng2 could be shown to serve as an indicator for monitoring disease activity in patients with OSCCs and other epithelial tumors [80,151].

To understand the complex circuit of Lng2-mediated OSCC progression, prospective clinical studies are warranted in order to investigate the relation between Lng2 reorganization, the structure of the invasive front, stromal activation, the expression and organization of extracellular oncofetal adhesion proteins, and EGFR downstream signaling in relation to the clinical outcome. This could be facilitated by the recent laboratory developments relating to spatial phenotyping and multiplex immunofluorescence staining. This could also pave the way for the development of novel Ln332-based targeted treatment strategies. Although there have been a few encouraging results using Ln332 functional blocking approaches, established laminin-based treatment options are not yet available [38].

Interestingly, in 1993, Noguchi and coworkers had already reported a comparative immunohistochemical study of Ln and CD3 positivity in OSCCs, demonstrating a relationship between Ln alterations, decreased T-cell tumor infiltration, and resistance to chemotherapy [152]. Recently, evidence has been provided supporting the ability of Lng2 to prevent T-cell infiltration and to attenuate the response to immune checkpoint inhibitors in non-small cell lung esophageal cancer. This effect depends on the upregulation of Lng2 synthesis by TGFβ1, which is, in turn, provided by stromal myCAF, emphasizing the impact of the TME on tumor biology and potential treatment strategies [39]. Therefore, Lng2-targeted therapeutic interventions must also be taken into consideration as part of combination therapies targeting EGFR signaling, immune checkpoint molecules, and others.

## Figures and Tables

**Table 1:** Overview of studies conducted to assess the diagnostic and prognostic value of the laminin γ2 chain expression pattern in oral squamous cell carcinomas.

Tissue Type	Method	Laminin γ2 Chain Pattern Analyzed	Relevance for Diagnosis/Prognosis	Reference
Dysplastic oral cells (brush biopsies)	Immunohistochemistry	Cytology, presence of positive tumor cells	Method-enhanced brush cytology with enhanced sensitivity	[77]
Preneoplastic oral lesions	Immunohistochemistry	Positive versus negative staining	Higher risk for tumor progression	[71]
Oral verrucous carcinoma versus well differentiated OSCC	Immunohistochemistry	Number of cytoplasmic positive tumor cells	>5% positive cells favors diagnosis of well-differentiated OSCCs	[78]
OSCC	Immunohistochemistry	Number of cytoplasmic positive tumor cells	Shorter survival period	[70]
OSCC	Immunohistochemistry	Immunohistochemical expression/4-point intensity scoring	Lng2: disease specific survival/4 gene signature including Lng2 predicts metastasis	[73]
OSCC	Genome expression profiling	Expression changes	Separation of invasive and metastatic OSCC	[20]
OSCC	Immunohistochemistry	Increased number of cytoplasmic positive tumor cells	Associated with tumor budding	[79]
OSCC	Immunohistochemistry	Extension of cytoplasmic tumor cell staining	Associated with aggressive growth patterns	[69]
OSCC versus high-risk oral lesions	Immunohistochemistry	Continuity of BM staining and basal versus suprabasal tumor cell staining	Associated with smoking and OSCC diagnosis	[72]
SCC from the tongue and floor of the mouth	Immunohistochemistry	Focal type versus diffuse type expression pattern	Independent factor for nodal metastasis	[74]
Tongue SCC	Immunohistochemistry	Number of cytoplasmic positive tumor cells	Decreased survival time	[66]
Tongue SCC	Immunohistochemistry	Peripheral versus diffuse cytoplasmic positive tumor cells	Decreased 3-year survival rate, increased cervical metastases	[67]
Tongue SCC	Transcriptome sequencing/Immunohistochemistry	Increased expression	Treatment failure in T1, T2/decreased disease free and overall survival	[68]
Head and neck SCC	Lng2 fragment ELISA	Changes in serum concentration	Monitoring clinical course and treatment results	[80]

## Abbreviations

3D	3-dimensional
aSMA	alpha smooth muscle actin
BM	basement membrane
CAF	cancer-associated fibroblast
ECM	extracellular matrix
EDA-Fn	EDA domain containing fibronectin
EDB-Fn	EDB domain containing fibronectin
EGF	epidermal growth factor
EGFR	epidermal growth factor receptor
EMT	Epithelial–mesenchymal transition
Fn	fibronectin
FNIII	fibronectin type III-like repeats
HD	hemidesmosome
HNSCC	head and neck squamous cell carcinoma
Ln	laminin
Ln332	laminin 332
Ln5	laminin 5
Lng2	laminin gamma 2 chain
MMP	matrix metalloproteinase
mRNA	messenger RNA
myCAF	cancer-associated fibroblast with a myofibroblast phenotype
oncf	oncofetal
oncfECM	oncofetal extracellular matrix
oncfFn	oncofetal fibronectin
oncfTn-C	oncofetal tenascin-C
OSCC	oral squamous cell carcinoma
RNA	ribonucleic acid
TGFβ1	transforming growth factor beta 1
TIMP	tissue inhibitor of metalloproteinases
TME	tumor microenvironment
Tn-C	tenascin-C
